# Verbal entrainment in autism spectrum disorder and first-degree relatives

**DOI:** 10.1038/s41598-022-12945-4

**Published:** 2022-07-07

**Authors:** Shivani P. Patel, Jennifer Cole, Joseph C. Y. Lau, Gabrielle Fragnito, Molly Losh

**Affiliations:** 1 Roxelyn and Richard Pepper Department of Communication Sciences and Disorders, Northwestern University, Evanston, IL USA; 2 Department of Linguistics, Northwestern University, Evanston, IL USA

**Keywords:** Psychology, Human behaviour, Social behaviour

## Abstract

Entrainment, the unconscious process leading to coordination between communication partners, is an important dynamic human behavior that helps us connect with one another. Difficulty developing and sustaining social connections is a hallmark of autism spectrum disorder (ASD). Subtle differences in social behaviors have also been noted in first-degree relatives of autistic individuals and may express underlying genetic liability to ASD. In-depth examination of verbal entrainment was conducted to examine disruptions to entrainment as a contributing factor to the language phenotype in ASD. Results revealed distinct patterns of prosodic and lexical entrainment in individuals with ASD. Notably, subtler entrainment differences in prosodic and syntactic entrainment were identified in parents of autistic individuals. Findings point towards entrainment, particularly prosodic entrainment, as a key process linked to social communication difficulties in ASD and reflective of genetic liability to ASD.

## Introduction

**Figure 1:**
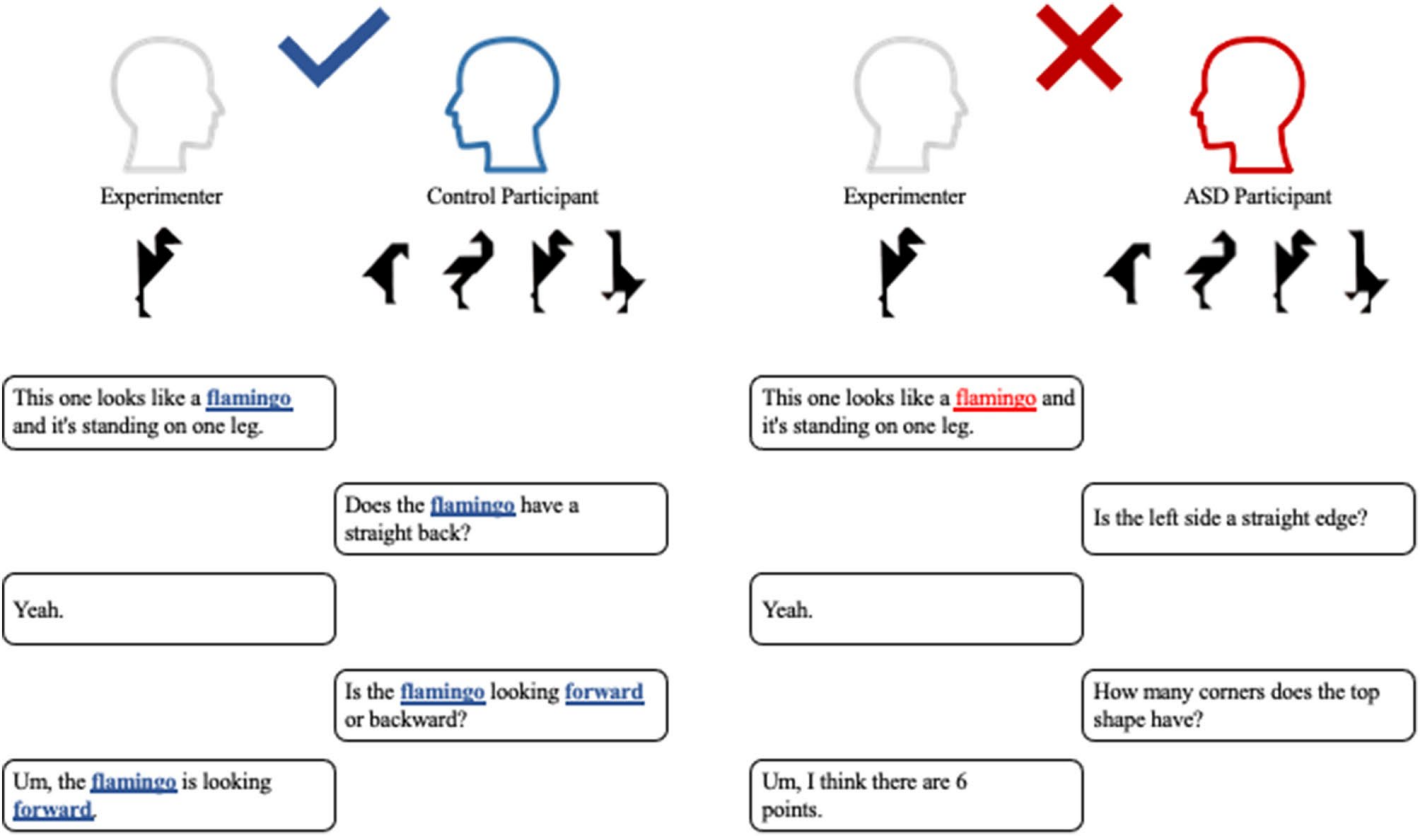
Schematic depicting lexical entrainment between an experimenter and a control participant (left) and an individual with ASD (right). On the left, the control participant uses the same terminology introduced by the experimenter (i.e., flamingo) and subsequently, the experimenter also uses the same terminology as the control participant (i.e., forward). This dyad exhibits lexical entrainment. On the right, the ASD participant uses distinct terminology (i.e., straight edge, corners) from that introduced by the experimenter (i.e., flamingo). There is a lack of lexical entrainment between the experimenter and ASD participant.

Connecting with others is an integral part of humans’ social drive. Entrainment, or the unconscious tendency to become more similar in speech or gesture to one’s communication partner, plays a key role in facilitating interpersonal connections. For instance, when one communication partner speaks at an increased rate, the other will often naturally increase their rate, too^1^. This increasing similarity (i.e., entrainment) between speakers not only supports establishment of rapport^2,3^ but is also predictive of relationship success^4^. Similarly, speakers may diverge in their language, such as when differentiating status (e.g., student vs. teacher), dialogue roles (e.g., explaining, vs. inquiring), or emphasizing varying points of view, which can be described as disentrainment. When effectively integrated, entrainment and disentrainment contribute to successful social interactions^5,6^. Disrupted entrainment or disentrainment may contribute to an array of social communication deficits, such as those evident in autism spectrum disorder (ASD). Impaired ability to develop and sustain social connections and fluent social communicative interactions is a hallmark of ASD, a genetically-based neurodevelopmental disorder characterized by the presence of repetitive behaviors and restricted interests^7^, as well as impairments in communication and distinct language domains, including prosody (e.g., intonation modulation^8^, volume modulation^9–11^, speech rhythm^12^ and rate^13^), lexico-semantics (i.e., word choice and meaning), and syntax (i.e., grammar)^7^. Entrainment across each of these language domains plays an important role in supporting the fluidity of social interactions and communication^14–20^, and when impaired can contribute to pervasive troubles in these areas (see Fig. 1 for schematic).

Evidence that subclinical traits associated with ASD often aggregate among first-degree relatives of individuals with ASD^21–23^, who do not display any clinical impairment, provides a potentially critical path for identifying links between observable traits and abilities, such as entrainment, and underlying neural and genetic factors that can inform the biological basis of these complex human traits and behaviors^24–26^. Indeed, studying the familiality and heritability of subclinical traits associated with a disorder is a powerful method for uncovering molecular genetic variation and neural circuitry implicated in heritable, but etiologically complex diseases (e.g., heart disease^27,28^ and diabetes^29^) and psychiatric conditions, such as schizophrenia^30,31^. Using this approach, studies of ASD have identified a Broad Autism Phenotype (BAP), which refers to a constellation of subclinical differences in social language and personality traits that mirror the core features of ASD in quality but are not associated with functional impairments^23,32–34^. Differences in language constitute a particularly significant component of the distilled expression of genetic liability to ASD in relatives, and entrainment is a potential contributor to such language differences. This study examined entrainment in ASD and in parents, across prosodic, lexical, semantic, and syntactic domains, using computational tools to objectively characterize entrainment. Across these domains, differences in parents of individuals with ASD have been identified in prosodic and lexical domains. More specifically, studies note differences in intonation and volume modulation, speech rate, and rhythm^21–23,33^, as well as use of overly formal language^21,23,33^. While few studies have examined semantic and syntactic differences in parents of autistic individuals, existing research suggests that skills in these domains are comparable to or perhaps exceed those of parent controls^35,36^.

Key language-related impairments in ASD, and the more subtle differences in parents, may importantly relate to entrainment. For instance, prosodic impairments in autistic individuals [*note:* Given expressed differences in preferences between identity-first and person-first language within the autism community, this manuscript alternates between the terms “individuals with autism” and “autistic individuals”] may influence entrainment mechanistically, where documented impairments in the coupling between auditory feedback (i.e., what one hears from oneself or one’s surroundings) and vocal motor commands (i.e., feedforward plan, or one’s motor plan to produce speech) in ASD undermine prosodic skills necessary for successful entrainment^37,38^. Differences in audio-vocal integration have also been documented in parents of individuals with ASD, suggesting that this critical process, and associated neural architecture related to speech processing, are influenced by ASD genetic vulnerability^37^. Verbal entrainment across linguistic domains is thought to require a parallel process to audio-vocal integration, such that listeners simulate heard speech input internally using a feedforward model, and the prediction error generated by this model influences listeners’ subsequent productions, yielding a production that is more similar to communication partners’ productions^39^. As such, inefficiencies in feedback and feedforward integration could impact verbal entrainment skills, with potentially far-reaching impact on social communication abilities in ASD.

In line with this suggestion, emerging evidence suggests that individuals with ASD do not exhibit speech rate entrainment^40^ and inconsistently entrain to measures of voice quality^41^. Entrainment along the lexical, semantic, and syntactic domains in ASD has also been implicated in relevant studies on priming, in which the initial use of a stimulus (e.g., word, phrase, concept) is thought to facilitate subsequent use of the stimulus by way of increased speed of neural activation of the stimulus^42^. While autistic individuals show typical effects of immediate lexical and semantic priming on simple picture-naming^43,44^ or fragmented-word^45^ tasks, these effects are dampened during extended timeframes^46,47^. Similarly, individuals with ASD exhibit comparable effects of lexical priming to their typically developing counterparts in picture-word naming^48^ and paradigms with long presentation durations of the prime and target, but no evidence of semantic priming in a lexical decision task^49^. Together, literature on lexical and semantic priming suggests impairments in entrainment may emerge during fast-paced, longer and less structured interactions, such as conversations, which are common in daily interactions, or semi-naturalistic collaborative games, such as the one used in the present study. Similarly, while some studies have shown that autistic individuals exhibit syntactic entrainment in highly structured contexts^17,50^, evidence that syntax is negatively impacted during conversation^51^ suggests further examination of syntactic entrainment in ASD is warranted.

This study utilized computational linguistic tools to objectively quantify prosodic, lexical, semantic, and syntactic entrainment among individuals with ASD, their parents, and respective control groups. We predicted that the autistic group would exhibit reduced entrainment across linguistic domains compared to controls. Given the subtle nature of language differences among parents of individuals with ASD, we predicted reduced entrainment in this group would be limited to prosodic and lexical domains, where listener ratings of language differences are readily apparent^21–23,33^. We predicted both parent groups would exhibit similar patterns of semantic and syntactic entrainment due to the lack of language differences in these domains^35,36^.

## Methods

### Participants

Twenty-three individuals with ASD (ASD group), 27 individuals with typical development (ASD Control group), 51 parents of individuals with ASD (ASD Parent group), and 31 parents of individuals with typical development (Parent Control group) participated in this study (Table 1). Inclusion criteria required that participants be native English speakers with no history of hearing loss, brain injury, presence of a known genetic condition other than ASD, or major psychiatric disorder. Additionally, individuals in either control group were excluded if they had first- or second-degree relatives with ASD or history of language related impairments. All autistic individuals had community diagnoses of ASD. Research-reliable examiners confirmed diagnoses using the Autism Diagnostic Observation Schedule-2nd Edition (ADOS-2)^52^ for all participants in the ASD and ASD Control groups.

**Table 1:** Participant demographics.

	ASD (*n* = 23)	ASD control (*n* = 27)	Group comparison (ASD vs. ASD control)	Effect size	ASD parent (*n* = 51)	Parent control (*n* = 31)	Group comparison (ASD parent vs. parent control)	Effect size
Sex, *Males:Females*	16:7	12:15	*X*^2^ (1, N = 50) = 3.18, *p* = 0.08	*V* = 0.25	18:33	5:26	*X*^2^ (1, N = 82) = 3.51, *p* = 0.06	*V* = 0.06
Chronological Age *M* (*SD*)	19.46 (6.13)*	15.45 (6.01)	*t*(48) = 2.33, *p* = 0.02	*d* = 0.66	50.07 (7.72)	47.02 (7.38)	*t*(80) = 1.77, *p* = 0.08	*d* = 0.40
ADOS-2 Autism Symptom Severity, *M* (SD)	8.35 (1.67)	1.31 (.62)	*t*(47) = 19.11, *p* < 0.001	*d* = 5.74				
Full scale IQ^a^, *M* (*SD*)	97.05 (18.74)**	119.62 (11.73)	*t*(45) = − 4.87, *p* < 0.001	*d* = − 1.47	112.96 (11.86)*	118.77 (9.89)	*t*(77) = − 2.27, *p* = 0.03	*d* = − 0.52
Verbal IQ^a,b^, *M* (*SD*)	97.30 (18.92)**	120.27 (11.37)	*t*(44) = − 5.04, *p* < 0.001	*d* = − 1.49	109.77 (11.71)	114.45 (10.21)	*t*(75) = − 1.78, *p* = 0.08	*d* = − 0.42
Performance IQ^a,b^, *M* (*SD*)	96.00 (21.81)**	116.46 (17.45)	*t*(44) = − 3.53, *p* < 0.001	*d* = − 1.04	112.70 (11.87)	117.47 (10.10)	*t*(75) = − 1.80, *p* = 0.08	*d* = − 0.42
Word count	599.04 (277.78)**	835.22 (199.60)	*t*(48) = − 3.49, *p* = 0.001	*d* = − 0.99	860.82 (240.41)*	996.39 (302.04)	*t*(80) = − 2.25, *p* = 0.03	*d* = − 0.51

* indicates p < 0.05 and ** indicates p < = 0.001.

^a^IQ measures were derived from the Wechsler Abbreviated Scale of Intelligence (WASI) for individuals 16 years of age or older and the Wechsler Intelligence Scale for Children-Fourth Edition (WISC-IV) for individuals younger than 16 years of age.

^b^Due to time constraints, some participants completed the 2-subtest version of the WASI or WISC-IV, which does not provide measures of Verbal and Performance IQ. The sample size for these measures is as follows: ASD n = 22; ASD Control n = 24; ASD Parent n = 48; Parent Control n = 29.

Intellectual functioning was assessed using the Wechsler Abbreviated Scale of Intelligence (WASI)^53^ for individuals 16 years of age or older and the Wechsler Intelligence Scale for Children-Fourth Edition (WISC-IV)^54^ for individuals younger than 16 years of age. Independent samples *t* tests revealed that the ASD group was significantly older (t = 2.33, p = 0.02) than the ASD Control group and had a significantly lower full scale IQ (t = − 5.01, p < 0.001), verbal IQ (t = − 5.04, p < 0.001), and performance IQ (t = − 3.53, p < 0.001) than the ASD Control group. Furthermore, the ASD group exhibited a significantly reduced word count overall compared to the ASD Control group (t = − 3.45, p = 0.001). The ASD Parent group did not differ significantly in chronological age (t = 1.77, p = 0.08) from the Parent Control group; however, they exhibited lower full scale IQ (t = − 2.23, p = 0.03), as well as marginal differences in verbal IQ (t = − 1.78, p = 0.08) and performance IQ (t = − 1.80, p = 0.08) compared to the Parent Control group. The ASD Parent group exhibited a significantly lower word count (t = − 2.25, p = 0.03) on the entrainment tangram task (described below) compared to parent controls.

Relationships between demographic variables of age, full scale IQ, verbal IQ, performance IQ, and word count with measures of entrainment were assessed using Pearson correlations. In the ASD and ASD control groups combined, increased age was associated with reduced lexical entrainment (r = − 0.37, p < 0.01) but not semantic (r = − 0.08, p = 0.56), syntactic (r = 0.15, p = 0.30), or prosodic entrainment (|r|s < 0.17, ps > 0.25). Higher full scale IQ was related to increased semantic entrainment in the ASD and ASD Control groups (r = 0.45, p = 0.001), which appears to be driven by performance IQ (correlation with semantic entrainment: r = 0.41, p < 0.01). Full scale IQ was not related to lexical (r = 0.23, p = 0.12), syntactic (r = 0.23, p = 0.12), or prosodic entrainment (|r|s < 0.03, ps > 0.05). Increased verbal IQ was related to greater semantic (r = 0.48, p < 0.001), syntactic entrainment (r = 0.46), p = 0.001), and prosodic entrainment of rhythm at the dialog act unit level factor 2 (syllable energy) (r = − 0.40, p < 0.01). Verbal IQ was not related to lexical entrainment (r = 0.15, p = 0.33) or remaining measures of prosodic entrainment (|r|s < 0.09, ps > 0.23). Increased performance IQ was related to greater lexical entrainment (r = 0.36, p = 0.02) but not syntactic (r = − 0.03, p = 0.84) or prosodic entrainment (|r|s < 0.10, ps > 0.07). Increased word count was related to greater semantic (r = 0.63, p < 0.001), syntactic (r = 0.32, p = 0.02), and prosodic entrainment on rhythm at the dialog act unit level factor 2 (syllable energy) (r = − 0.34, p = 0.02). Word count was not related to lexical (r = 0.13, p = 0.36) entrainment nor remaining measures of prosodic entrainment (|r|s < 0.14, ps > 0.36).

In the ASD Parent and Parent Control groups, age was not related to lexical (r = − 0.07, p = 0.55), semantic (r = − 0.08, p = 0.50), syntactic (r = 0.08, p = 0.49), or prosodic entrainment (|r|s < 0.11, ps > 32). Higher full scale IQ was related to increased lexical entrainment (r = 0.29, p < 0.01), which appears to be driven by verbal IQ (correlation with lexical entrainment: r = 0.27, p = 0.02). Full scale IQ and verbal IQ, respectively, were not related to semantic (r = 0.15, p = 0.19; r = 0.14, p = 0.24), syntactic (r = 0.11, p = 0.34; r = 0.20, p = 0.08), or prosodic (|r|s < 0.05, ps > 0.27; |r|s < 0.11, ps > 0.28) entrainment. Performance IQ was not related to lexical (r = 0.20, p = 0.09), semantic (r = 0.11, p = 0.40), syntactic (r = − 0.002, p = 0.99), or prosodic entrainment (|r|s < 0.08, ps > 0.19). Increased word count was related to greater semantic entrainment (r = 0.29, p < 0.01) and prosodic entrainment on F0 at the salient syllable level factor 1 (F0 trends) (r = − 0.23, p = 0.04). Conversely, increased word count was related to reduced prosodic entrainment on F0 at the salient syllable level factor 2 (F0 envelope) (r = 0.68, p < 0.001).Word count was not related to lexical (r = 0.16, p = 0.15) entrainment nor remaining measures of prosodic entrainment (|r|s < 0.05, ps > 0.54).

### Entrainment tangram task

**Figure 2:**
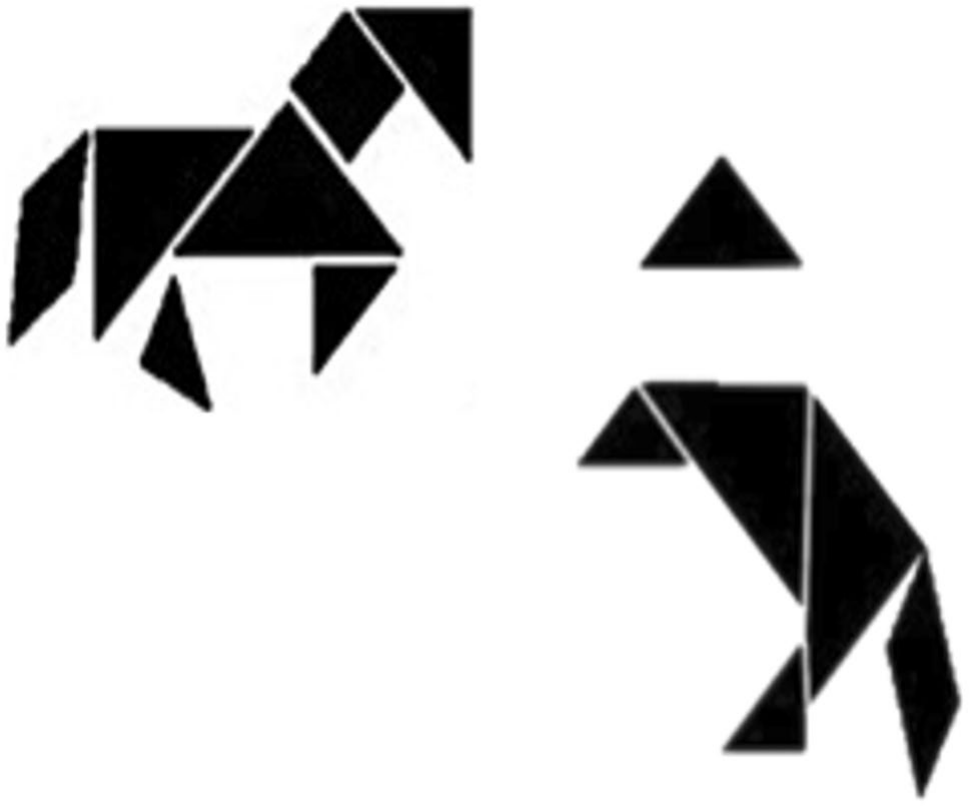
Tangram silhouettes.

Each participant played a collaborative game^55^ with one of two trained examiners. The examiner and the participant were both given a packet of tangram silhouettes that only they could see (see Fig. 2 for an example) during the task. During each round of the game, one partner viewed a page containing one tangram silhouette while the other partner viewed a page with four tangram silhouettes, one of which had an arrow pointing to it. The game required the partners to converse in order to determine if the silhouette described by the partner who was viewing the page that contained only one image matched the silhouette with the arrow pointing to it on the other partner’s page. Upon coming to a decision regarding whether or not the silhouettes matched, the partners verified their decision by showing each other the silhouettes. Regardless of whether or not the partners reached a correct or incorrect decision, they alternated roles for a minimum of six times and played the game for a total task duration of 10–15 min. To reduce variability in examiner influence on entrainment, the two examiners utilized semi-scripted responses and prompts for each silhouette.

During the task, the participant and examiner each wore a head-mounted microphone (Audio-Technica System 10 HS Sys w/92cW-TH), which recorded speech to separate channels. The conversations were manually text-transcribed using ELAN^56^ software and word count was calculated based on the participant’s transcribed speech. Given differences in prosody based on communicative intent (e.g., question vs. statement), all utterances were manually categorized using a dialog tag set developed for spontaneous task-oriented spoken dialogues^57^ to allow for analysis of prosodic entrainment within discourse segments with the same communicative intent. The dialog tag set distinguishes utterances based on their discourse goal. Importantly, dialog acts were determined solely based on the transcribed utterances. Incomplete or abandoned utterances were excluded from analyses as they were unable to be assigned a dialog act tag. Fifteen percent of all files were transcribed and dialog act tagged by a second individual. Average word-word reliability was 95.82%. Fleiss' kappa was used to assess agreement between raters’ dialog act tagging over and above chance agreement, and showed that there was good agreement between raters, κ = 0.664, p < 0.0005.

### Prosodic entrainment

Measures of prosodic entrainment were derived using the contour-based, parametric, and superpositional intonation stylization (CoPaSul)^58^ toolkit, which allows for description of global (measured at the level of the labeled dialog act unit) and local (measured at the level of a salient syllable) pitch/F0 contours parametrically in terms of polynomial coefficients. Prosody measures from CoPaSul draw on a range of acoustic measures related to pitch, intensity, and rhythm, computed within analysis windows corresponding to syllables and dialog acts, which may be important for entrainment. See Supplementary Table 1 for a detailed list of each of the acoustic measurements extracted in the present study. F0 was extracted using autocorrelation in Praat (version 6.1.06) with a sample rate of 100 Hz. Energy in terms of root mean squared deviation of the amplitude of the speech waveform within the analysis window was calculated with the same sample rate as F0 in Hamming windows of 50 ms length. Rhythm was measured as the number of salient syllables per second and the influences of the salient syllable level on the F0 and energy contours, where a salient syllable is automatically detected as exceeding threshold levels of energy and duration, corresponding to phrase/sentence level prominence (stress)^58,59^.

Prosodic entrainment was assessed at the dialog act level, a phrase or sentence that expresses the speaker’s communicative intention in a conversational interaction (e.g., a query, reply, or explanation), and the salient syllable level, which corresponds to the perceptually salient stressed syllable of a word (see^60^ for additional details). English uses prosodic distinctions at the salient syllable level to encode information structure (e.g., prosodic enhancement or “accenting” of words that answer a question (i.e., focused words) or that add new information to the discourse). Prosodic marking of dialog act and information structure aids the listener in integrating the current utterance with prior discourse context and with tracking the advancement of conversational goals. Prosodic encoding of discourse meaning (dialog act, information structure) is manifest in the acoustic signal primarily through pitch patterning, measured in terms of fundamental frequency (F0) and the co-variation of pitch and acoustic energy (rhythm). Accordingly, this study examined evidence of entrainment in measurements related to pitch/F0 in dialog act units and salient syllables, as well as rhythm in dialog act units. A factor analysis was used to reduce the large number of pitch/F0 and rhythm measurements calculated for prosodic entrainment in both measurement domains.

For each dialog act segment for a given speaker, four random samples with replacement of 1000 were drawn. The parameters of each sample were as follows: (1) same dyad, same dialog act; (2) across dyads, same dialog act; (3) same dyad, across dialog acts; (4) across dyads, across dialog acts. Sampling was conducted separately for child and parent groups, inclusive of diagnostic group. Pairings across dyads are considered to provide a control baseline against which entrainment can be measured and is referred to as a “surrogate” conversation. Pairings within the same dyad reflect the “real” conversation participants engaged in. Entrainment was measured by the absolute distance between the respective speakers’ value on a given variable from the mean value of the variable. Thus, smaller values reflect greater entrainment. Variables were extracted using the parameters outlined in the CoPaSul manual^58^.

Given the large number of acoustic variables that may contribute to prosodic entrainment, we conducted a series of exploratory factor analyses (EFA) using the factoextra^61^ and nFactors^62^ packages for R statistical software, in order to identify implicit variables underlying the variables measured by CoPaSul and thus reduce the number of variables included in the analyses. As such, separate EFAs were conducted for the following: (1) fundamental frequency measures extracted from the dialog act unit; (2) fundamental frequency measures extracted from the salient syllable; (3) rhythm measures extracted from the dialog act unit level. EFAs were run with a promax rotation, which is an oblique rotation that allows for correlated factors. For each EFA, the number of factors was determined using the Kaiser criterion, which indicates that factors with eigenvalues greater than 1 should be included, and through inspection of scree plots to determine the number of factors after which the eigenvalues make a sharp drop. Based on these criteria, each of the EFAs in the ASD and ASD Control groups, as well as in the parent groups, resulted in a 2-factor model. Subsequently, a series of confirmatory factor analyses (CFA) were run for each of the three levels noted above using the groupings derived from the EFA. Factor loadings from the CFA are indicated in Supplementary Table 2. CFA scores were derived for each participant, yielding a total of 6 prosodic entrainment variables which were used in subsequent analyses of prosodic entrainment.

### Lexical, semantic, and syntactic entrainment

Measures of lexical, semantic, and syntactic entrainment were extracted using the open source Python library Analyzing Linguistic Interactions with Generalizable techNiques (ALIGN)^63^. In the initial phase of ALIGN processing, the data are automatically cleaned and standardized such that contiguous utterances are transformed into turns so that each transcript uniformly alternates between each speaker. Additionally, a part-of-speech tag was generated for everything said in a given turn. Subsequently, a random pairing of speakers from different dyads was created for each conversation to create a control baseline, referred to as a surrogate conversation. In the second phase of ALIGN, scores for lexical, syntactic, and semantic entrainment were generated for each turn-by-turn exchange in both the real and control baseline (“surrogate”) interactions. Importantly, ALIGN captures the directionality of utterances between interlocutors, allowing for analysis of the participant entraining to the examiner and vice-versa. Given the present study’s focus on characterizing entrainment in ASD, analyses focused solely on values derived for utterances in which the participant responded to the examiner. Lexical entrainment was based on lemmatized words. A lemmatized word is the root form of a word. For example, the words “runs,” “running,” and “ran” are forms of the root word “run,” which is the lemma of these words. Semantic entrainment was based on Word2Vec^64^ representations of the corpus and syntactic entrainment on bigrams of part-of-speech tags. Bigrams of part-of-speech (POS) tags refer to two adjacent labels denoting the part of speech within a speaker’s utterance. For example, in the phrase “It looks like a bird” the bigrams of POS tags would be [“pronoun verb”] [“verb preposition”] [“preposition determiner”] [“determiner noun”]. Lexical and syntactic entrainment scores resulted in a score ranging from 0 to 1, with higher scores reflecting greater alignment. Semantic scores range from − 1, reflecting completely opposite semantic content, to 1, reflecting identical semantic content.

### Statistical analysis

Prosodic, lexical, syntactic, and semantic entrainment were analyzed using a series of mixed effects linear regression models conducted using the lme4 package^65^ for R statistical software. Separate models were conducted to examine differences in the ASD vs. ASD Control groups and the ASD Parent vs. Parent Control groups. Models investigating prosodic entrainment included main effects of conversation type (real vs. surrogate), dialog act pairing (same dialog act between speakers vs. different), and group, as well as all interaction terms. Models for lexical, semantic, and syntactic entrainment included a main effect of conversation type (real vs. surrogate), time (turn in conversation), and group, as well as all interaction terms. Additionally, models assessing lexical, semantic, and syntactic entrainment controlled for participant word count and included by-participant random intercepts, as well as random slopes corresponding to all fixed effects. Models did not control for measures of IQ as they did not relate to outcome measures in the present study. See Supplementary Tables 3 and 4 for a full summary of statistical findings. Additional analysis of relationships between measures of verbal entrainment and social communication skills, as well as within-family associations are reported in Supplementary Methods and Supplementary Results. Brief interpretation of additional analysis is included in the Supplementary Discussion.

### Ethical approval

Ethical approval for this study was obtained by the IRB of Northwestern University and all research was performed in accordance with relevant guidelines and regulations.

### Informed consent

Written informed consent was obtained from each study participant and/or a parent or legal guardian.

## Results

For ease of interpretation, only overall effects of entrainment and interactions between entrainment and group are reported in the text (see Table 2 for a visual summary). Supplementary Tables 3 and 4 detail remaining effects and interaction terms.

**Table 2:**
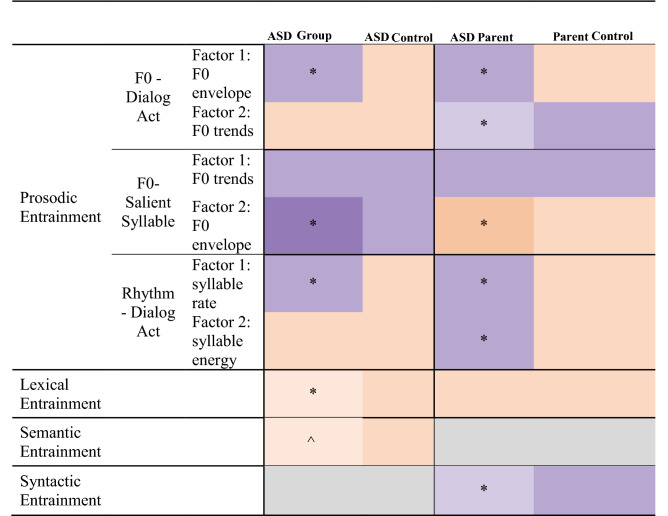
Summary of entrainment findings across groups. The colored scale indicates a main effect of conversation type (real vs. surrogate), with orange reflecting entrainment within a group and purple reflecting disentrainment within a group. Gray denotes domains in which there was not a significant main effect of conversation type. * indicates a significant (*p* < 0.05) interaction between conversation type and group, thereby reflecting a difference between the ASD vs. ASD Control groups or ASD Parent vs. Parent Control groups. *^* indicates a marginal (*p* = 0.05) interaction between conversation type and group.

### Verbal entrainment in ASD

#### Prosodic entrainment

**Figure 3:**
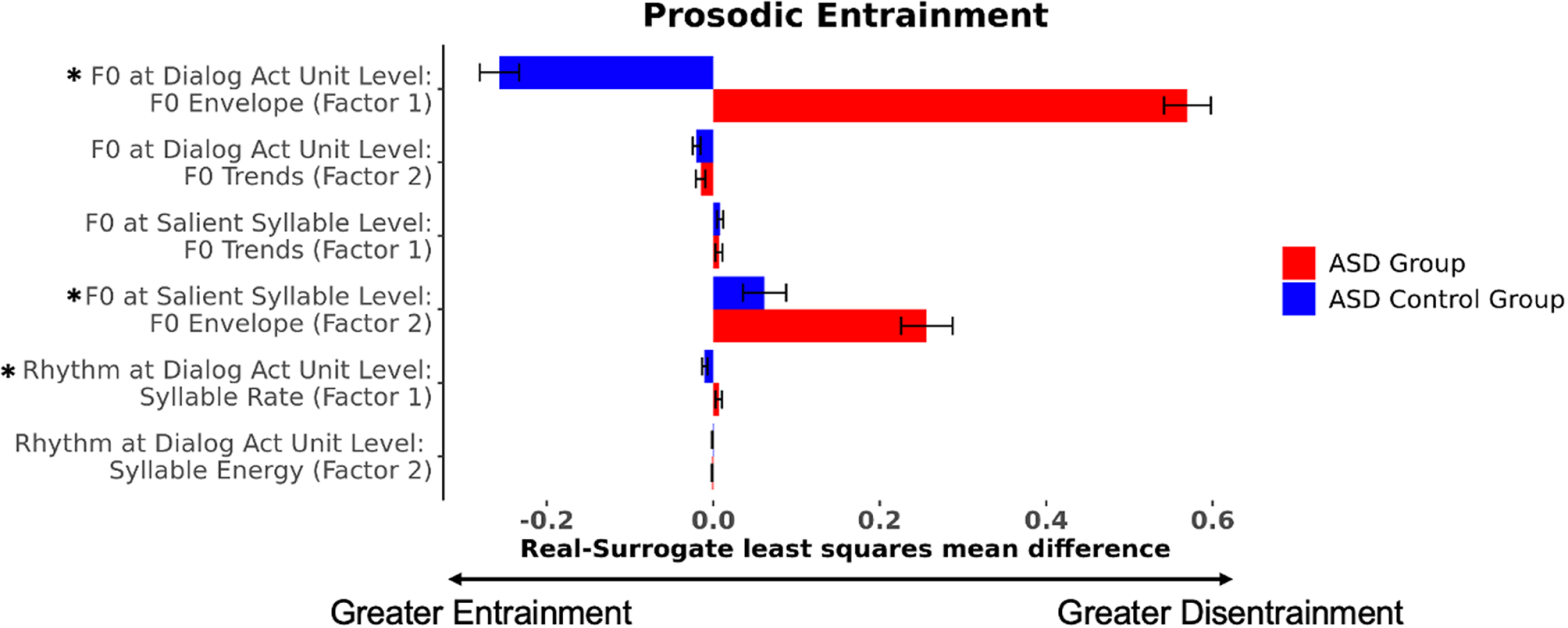
The ASD group exhibited disentrainment in measures of the F0 envelope (factor 1) in dialog act units compared to entrainment in the ASD Control group. At the salient syllable level, both groups exhibited disentrainment in the F0 envelope in salient syllables (factor 2), though disentrainment was greater for the ASD group, indicating a greater resistance to converge with their partner in the scaling of F0 movements. The ASD group exhibited rhythmic disentrainment (factor 1—related to salient syllable rate) compared to entrainment in the ASD Control group. * indicates a statistically significant (*p* < 0.05) difference between the ASD and ASD Control groups. Error bars depict standard error.

Individuals with ASD exhibited disentrainment in measures of the F0 envelope (factor 1) in dialog act units (β = 0.83, p < 0.001), indicating that they diverged from their conversation partners in the scaling of F0 movements marking dialog act, whereas the ASD Control group exhibited entrainment for the same factor (Fig. 3). Both groups exhibited entrainment in measures of dynamic F0 trends (factor 2) in dialog act units (β = − 0.02, p < 0.001), converging with their conversation partner in the dynamic pitch patterns used to mark dialog act distinctions. In the smaller domain of the salient syllable, both ASD and ASD Control groups showed similar effects of disentrainment in dynamic F0 trends (factor 1) (β = 0.007, p = 0.007), diverging from their conversation partners in the pitch patterns marking information structure distinctions. Differences between the groups were observed in measures of the F0 envelope in salient syllables (factor 2). Both groups demonstrated disentrainment of this factor, though with a greater degree of disentrainment evident in the ASD group (β = 0.20, p < 0.001), indicating a greater resistance to converge with their partner in the scaling of F0 movements. The ASD group exhibited rhythmic disentrainment on syllable rate (factor 1) compared to entrainment for controls (β = 0.02, p < 0.001). However, across groups similar effects of rhythmic entrainment were observed on syllable energy (factor 2) (β = − 0.001, p < 0.001).

#### Lexical, semantic, and syntactic entrainment

**Figure 4:**
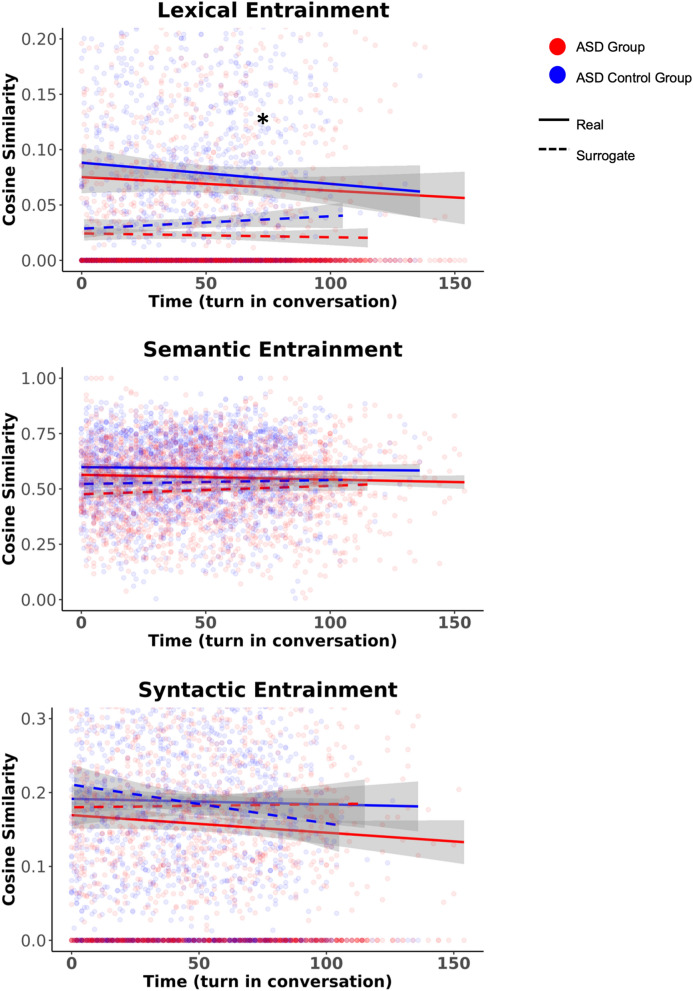
The ASD group exhibited reduced lexical entrainment compared to the Control group. Differences in semantic entrainment between groups approached significance (p = 0.05) The ASD and ASD Control groups did not differ on syntactic entrainment. * indicates a statistically significant (*p* < 0.05) difference between the ASD and ASD Control groups.

The ASD group exhibited reduced *lexical* entrainment compared to their control counterparts (β = 0.64, p = 0.02; Fig. 4). While *semantic* entrainment was evident in the ASD and ASD Control groups (β = 0.33, p = 0.01), the ASD group exhibited marginally reduced entrainment over the course of the interaction (β = − 0.01, p = 0.05). There were no statistically significant effects of *syntactic* entrainment (β = 0.08, p = 0.54).

### Verbal entrainment in parents of individuals with ASD

#### Prosodic entrainment

**Figure 5:**
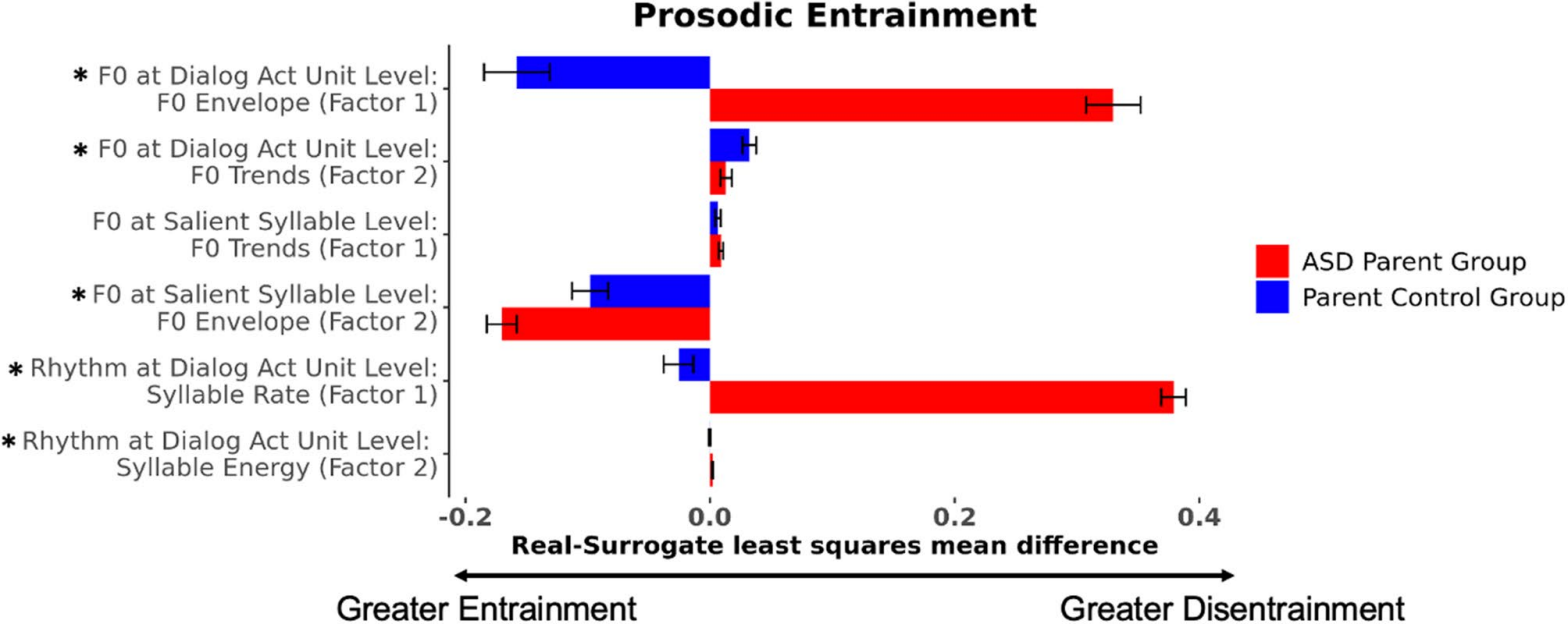
The ASD Parent group exhibited disentrainment in the F0 envelope in dialog act units (factor 1) and the second factor of rhythm in dialog act units compared to entrainment in the Parent Control group. Both parent groups exhibited disentrainment in dynamic F0 trends (factor 2) at the dialog act unit level, though the ASD Parent group exhibited reduced disentrainment relative to controls. Conversely, the ASD Parent group exhibited greater entrainment on the F0 envelope (factor 2) at the salient syllable level compared to the Parent Control group. * indicates a statistically significant (*p* < 0.05) difference between the ASD Parent and Parent Control groups. Error bars depict standard error.

On *prosodic* entrainment in the F0 envelope in dialog act units (factor 1), results revealed disentrainment in the ASD Parent group compared to the Parent Control group (β = 0.49, p < 0.001; Fig. 5). While parent groups overall exhibited disentrainment in dynamic F0 trends (factor 2) at the dialog act unit level, the ASD Parent group exhibited reduced disentrainment relative to controls (β = − 0.02, p = 0.01). For dynamic F0 trends (factor 1) at the salient syllable level, similar disentrainment was evident across both parent groups (β = 0.008, p < 0.001). For the F0 envelope (factor 2) at the salient syllable level, the ASD Parent group exhibited greater entrainment compared to the Parent Control group (β = − 0.07, p < 0.001). For syllable rate (factor 1; β = 0.40, p < 0.001) and syllable energy (factor 2; β = 0.002, p = 0.001) at the dialog act unit level, the ASD Parent group exhibited disentrainment compared to patterns of entrainment among the Parent Control group.

#### Lexical, semantic, and syntactic entrainment

**Figure 6:**
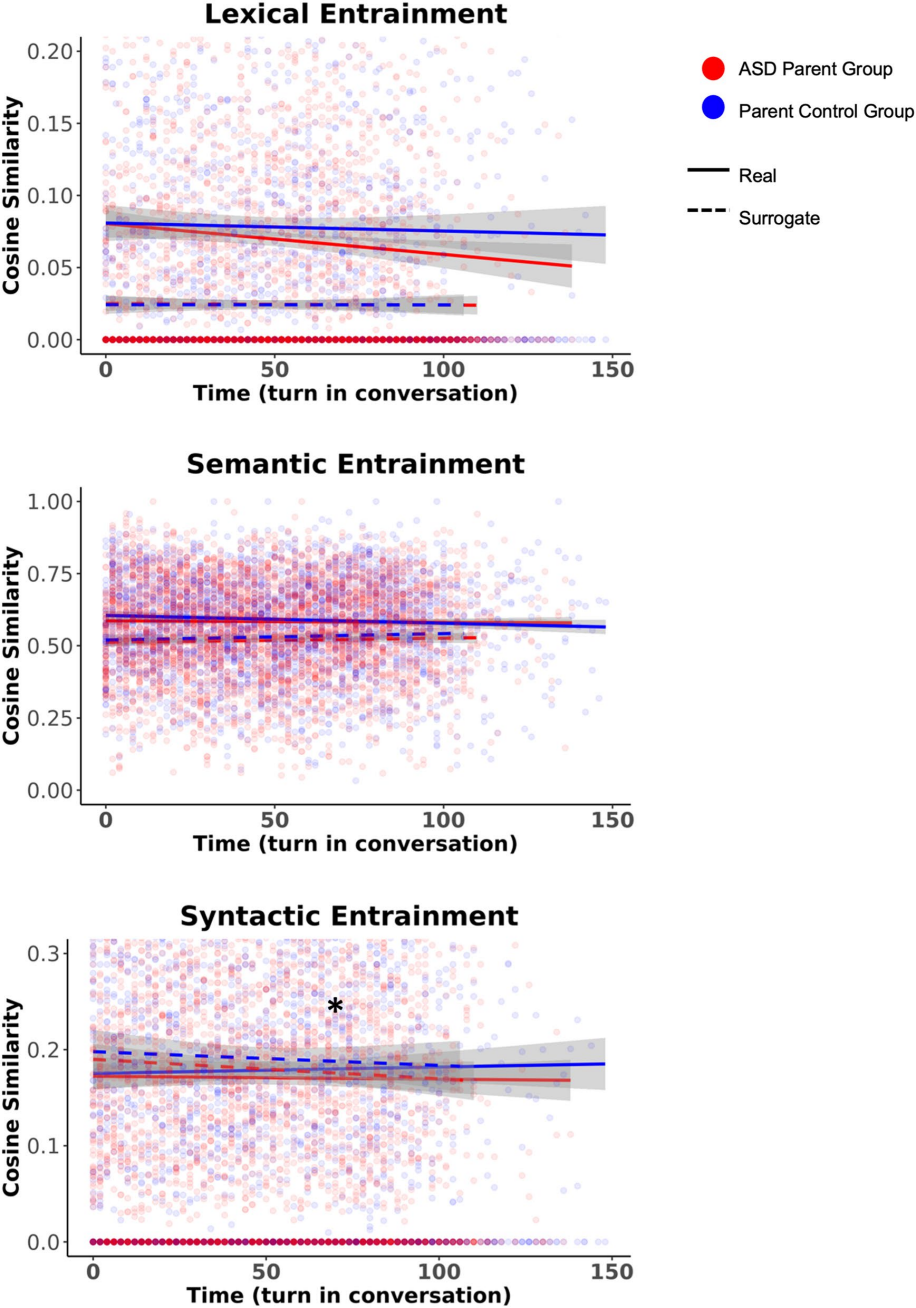
The ASD Parent group did not differ from the Parent Control group in lexical or semantic entrainment. The ASD Parent group exhibited reduced syntactic entrainment compared to the Parent Control group. * indicates a statistically significant (*p* < 0.05) difference between the ASD Parent and Parent Control groups.

Overall, the parent groups exhibited *lexical* entrainment (β = 0.33, p < 0.01; Fig. 6). No evidence of *semantic* entrainment nor disentrainment was detected in the parent groups (β = − 0.03, p = 0.80). The ASD Parent group exhibited reduced *syntactic* disentrainment compared to the Parent Control group (β = 0.53, p = 0.02).

## Discussion

This study aimed to assess verbal entrainment across prosodic, lexical, semantic, and syntactic entrainment in individuals with ASD and their parents compared to respective control groups. We predicted that the autistic group would exhibit reduced entrainment across linguistic domains compared to controls. Given the subtle nature of language differences among parents of individuals with ASD, we predicted reduced entrainment in this group would be limited to prosodic and lexical domains, where language differences in parents of individuals with ASD have been previously documented. Robust differences in entrainment across prosodic and lexical domains were evident in autistic individuals. Parallel differences in prosodic entrainment were evident among parents of individuals with ASD and are particularly striking considering the lack of any clinical impairment in this group. Contrary to our predictions, parents of autistic individuals exhibited differences in syntactic entrainment.

In ASD, distinct patterns of prosodic and lexical, but not semantic nor syntactic, entrainment emerged. Within the domain of prosody, autistic individuals exhibited increased disentrainment (i.e., divergence between conversational partners) rather than entrainment, whereas controls primarily exhibited entrainment and only minimal disentrainment. Considering evidence that positive perceptions of social interactions are related to the effective integration of entrainment and disentrainment^5,6,66–70^, it is perhaps unsurprising that patterns of entrainment and disentrainment were evident across groups. For instance, consistent entrainment (in the absence of disentrainment) throughout an interaction may be negatively interpreted as mockery or contribute to a sense of false flattery; meanwhile, effective integration of entrainment and disentrainment may facilitate more successful, naturalistic interactions. However, the present findings implicate breakdowns in typical entrainment (and disentrainment) patterns as key contributors to the social communication deficits in ASD.

More specifically, prosodic disentrainment was apparent in multiple domains of measurement (dialog act unit and salient syllable) in individuals with ASD, whereas controls exhibited disentrainment exclusively at the salient syllable level. Across both levels of measurement in the ASD group, greater disentrainment was evident on the factors providing information about the F0 envelope, such as the F0 mean and max, rather than information related to dynamic F0 trends in the speech signal indexed by variables such as slope and RMSD of the baseline, midline, and topline of the F0 contour. This suggests that rather than an overall deficit in prosodic entrainment of F0/pitch, autistic individuals exhibit a specific deficit related to entrainment on measurements of F0 scaling. Differences in these acoustic properties play important roles in a variety of prosodic functions. For instance, prior work has identified patterns of increased and decreased mean F0, as well increased maximum F0 in individuals with ASD, on structured tasks assessing affect expression (e.g., conveying a target emotion), contrastive focus (e.g., the WHITE cow vs. the white COW), as well as expression of dialog act distinctions at the end of a conversational turn (e.g., producing a statement vs. question) among others. Indeed, mean and maximum F0 (as well as duration) were strong predictors of naïve listeners’ ratings of prosodic atypicalities in individuals with ASD^71^. These findings extend this work by demonstrating the broader impact these components of the speech signal can have on ongoing interactions. Beyond the scope of disrupting specific prosodic functions, it appears that the same components hinder entrainment for autistic individuals and their communication partners.

Additionally, the ASD group showed disentrainment in rhythm (factor 1—syllable rate) in the larger span of the dialog act, suggesting a role for rhythmic entrainment in social communication difficulties in ASD. This finding extends a prior report of problematic rhythmic entrainment in adults with ASD, showing that adults with ASD had difficulty entraining speech rate to a digitally manipulated confederate’s speech (although disentrainment was not examined)^40^. Results also expand upon prior findings of speech rate or rhythm atypicalities in individuals with ASD^12,13^, by delineating a mechanism through which these differences impact social interactions. Despite disentrainment on the first factor of rhythm, autistic individuals exhibited comparable entrainment to controls on the second factor, which included variables reflecting the influence of syllables on the energy contour of each dialog act unit. As such, rhythmic entrainment, similar to F0/pitch entrainment discussed above, appears to be complexly impacted in ASD.

Individuals with ASD also exhibited reduced lexical entrainment despite intact overall semantic entrainment. This suggests that while autistic individuals aligned with their communication partner on overall message content, key terminology may have differed. However, individuals with ASD demonstrated marginally reduced semantic entrainment over the course of the interaction, which is consistent with studies of semantic priming that have demonstrated diminished effects with increased duration of the prime and target^45,48,49^. It is perhaps unsurprising that syntactic entrainment was not detected in the ASD nor ASD Control groups, given prior findings of dampened effects of syntactic entrainment during ongoing interactions, whereas other domains of verbal communication (i.e., prosodic, lexical, semantic) and related factors may require more cognitive resources, leading to divergent, diminished, or absent syntactic entrainment^44,66^. It is also possible, however, that other contexts allowing for more extended language exchange opportunities may be better suited for examining syntactic entrainment.

Importantly, differences in prosodic and syntactic entrainment were detected among parents of autistic individuals. Of note, parents of individuals with ASD did not differ in lexical entrainment as predicted. Given that lexical differences in parents of autistic individuals have primarily been identified in conversational tasks^21,23,33^, it is possible that the semi-structured nature of the task used in this study obscured possible differences in lexical entrainment by limiting the type of vocabulary used to simple descriptions of images (e.g., shapes, animals, objects), rather than the greater variety of lexical items that may be used in free flowing conversation. Nevertheless, as in ASD, parents exhibited prosodic disentrainment on the factor reflecting F0 envelope measurements, such as mean and max F0, at the dialog act unit level, whereas parent controls exhibited entrainment. This parallel finding in ASD and parents supports prior work showing differences in prosody in both ASD and among first-degree relatives and points toward differences in this element of prosodic entrainment as a potential marker of genetic liability to ASD. Such differences in entrainment are certainly not the result of genetics alone but rather the complex interplay between genetic susceptibility to ASD and environmental factors known to influence communication skills^72^. However, further patterns of differences in prosodic entrainment were more complexly expressed across ASD and ASD parent groups. Contrary to findings in individuals with ASD, parents of individuals with ASD exhibited greater entrainment on F0 envelope measures at the salient syllable level compared to parent controls. Together, findings across measurement levels revealed both elevated prosodic disentrainment and entrainment, which may reflect less effective integration of these processes, and contribute to the subtle pragmatic language differences noted in first-degree relatives of autistic individuals^21,23,33^. Findings of increased rhythmic disentrainment (assessed at the dialog act unit level) in parents provide further evidence linking increased disentrainment to broader pragmatic language differences noted at the level of a communicative intention.

Syntactic disentrainment was evident among both parent groups and is consistent with evidence challenging generalizations of syntactic priming/entrainment effects identified in structured laboratory-based studies to conversational contexts^44,66^. In line with prior work^66^, syntactic disentrainment may be a reflection of successful conversations in which lexical and semantic properties are imitated using *distinct* syntactic structures to serve a variety of functions, such as reformulating an interlocutor’s statement into a question, elaborating, correcting an interlocutor, or making a joke. Though unexpected, reduced syntactic disentrainment detected among parents of individuals with ASD may index reduced effectiveness in achieving the full spectrum of these functions, and therefore, have a large impact on broader pragmatic language abilities.

In sum, findings point to differences in prosodic entrainment in both autistic individuals and their parents, and broader verbal entrainment difficulties in ASD across lexical and semantic domains of communication, suggesting that entrainment may be an important process contributing to the social communication deficits characteristic of ASD and subclinical social communication styles associated with genetic liability to ASD. Findings additionally demonstrate the feasibility of applying interdisciplinary, open-source computational tools to research focused on clinical populations to promote reproducibility and efficiency by reducing variation across manual coding systems and the time required to apply such systems. This is of critical importance in ASD research given the breadth of clinical heterogeneity observed, where removing variability inherent to differences in coding schemes and subjectivity of human raters may yield a clearer understanding of the true variability in ASD and aid in stratification of more phenotypically and etiologically homogeneous subgroups.

The present findings should be considered with some limitations in mind. Considering the heterogeneous presentation of ASD, there is likely individual variability in patterns of entrainment across autistic individuals that should be explored in future work. Such variability may be related to intrapersonal factors, such as language skills^73,74^, word count/utterance length, cognitive abilities, age, and sex. Cognitive abilities and age were taken into consideration by investigating relationships between intellectual functioning, age, and measures of entrainment. In the ASD and ASD Control groups, lower IQ, specifically performance IQ, and increased age were associated with reduced lexical entrainment. It is possible that these confounding variables underlie differences in lexical entrainment observed between the ASD and ASD Control groups. Greater cognitive abilities, namely nonverbal cognitive abilities, may facilitate lexical entrainment during interactions with an unfamiliar communication partner. Of note, however, both groups exhibited mean full scale, verbal, and performance IQs within the normal range. Reduced lexical entrainment with increased participant age is surprising considering research documenting increased entrainment among speakers who share more similarities^75^, and in this case, older participants would have been closer in age to the examiner. Further research is necessary to clarify the roles of cognitive ability and age in lexical entrainment. Importantly, cognitive abilities and age were not related to variables in which prosodic entrainment differences were detected in the ASD and ASD Parent groups, further highlighting prosodic entrainment as a key area impacting social communication skills in ASD. Nonetheless, individuals with a wider range of cognitive abilities, language levels, ages, as well as larger sample of autistic females, should be included in future work to examine verbal entrainment in an ecologically valid sample of autistic individuals. Several studies have demonstrated distinct clinical presentation between males and females with ASD, including apparently linguistically-mediated camouflaging of symptoms among females^76–78^, that could complexly interrelate with entrainment skills. Recent investigations suggest that conversational rapport also varies with interpersonal factors, such that rapport is higher among dyads matched on neurotypes (e.g., autistic–autistic or neurotypical–neurotypical) rather than mixed neurotype (e.g., autistic–neurotypical)^79–81^. Given the strong relationships between rapport and entrainment^2,3^, neurotype matching differences across dyads may present an alternative explanation for the present findings and should be investigated in future research. Importantly, this may contribute to the surprising amount of variation evident among the surrogate dyad pairings. Alternative explanations for this variability may also include differences in the types of dialog acts (e.g., yes/no question, reply, acknowledgement) used by autistic individuals and their parents compared to respective controls. It will be important for future work to further investigate such differences and perhaps provide an alternative method for generating a more consistent baseline condition. Future work may also examine changes in verbal entrainment in response to intervention, as well as determining the most fruitful interventions to support verbal entrainment. For example, addressing deficits at higher levels of the linguistic hierarchy, such as lexical entrainment, may yield the most immediate benefits to broader social communication skills. Moreover, interventions may vary greatly from targeting specific repair strategies to improve entrainment within a given domain or targeting deficits in naturally occurring situations.

## Supplementary Information

Supplementary Information.

## Data Availability

Data used in the preparation of this manuscript will be shared with the NIH-supported National Database for Autism Research (NDAR). This manuscript reflects the views of the authors and may not reflect the opinions or views of the NIH.

## References

[1] Manson JH, Bryant GA, Gervais MM, Kline MA (2013). Convergence of speech rate in conversation predicts cooperation. Evol. Hum. Behav..

[2] Pardo JS (2006). On phonetic convergence during conversational interaction. J. Acoust. Soc. Am..

[3] Semin, G. R. & Cacioppo, J. T. Grounding social cognition: Synchronization, coordination, and co-regulation. *Embodied Grounding Soc. Cogn. Affect. Neurosci. Approaches* 119–147 (2008).

[4] Ireland ME (2011). Language style matching predicts relationship initiation and stability. Psychol. Sci..

[5] De Looze C, Scherer S, Vaughan B, Campbell N (2014). Investigating automatic measurements of prosodic accommodation and its dynamics in social interaction. Speech Commun..

[6] Perez, J., Galvez, R. & Gravano, A. Disentrainment may be a positive thing: A novel measure of unsigned acoustic-prosodic synchrony, and its relation to speaker engagement. in *Proceedings of Interspeech* 1270–1274 (2016).

[7] American Psychiatric Association (2013). Diagnostic and Statistical Manual of Mental Disorders.

[8] Asghari SZ, Farashi S, Bashirian S, Jenabi E (2021). Distinctive prosodic features of people with autism spectrum disorder: A systematic review and meta-analysis study. Sci. Rep..

[9] Shriberg L, Paul R, McSweeny J, Klin A, Volkmar FR (2001). Speech and prosody characteristics of adolescents and adults with high-functioning autism and Asperger syndrome. J. Speech Lang. Hear. Res..

[10] Pronovost W, Wakstein MP, Wakstein DJ (1966). A longitudinal study of the speech behavior and language comprehension of fourteen children diagnosed atypical or autistic. Except Child..

[11] Hubbard DJ, Faso DJ, Assmann PF, Sasson NJ (2017). Production and perception of emotional prosody by adults with autism spectrum disorder. Autism Res..

[12] Paul R, Bianchi N, Augustyn A, Klin A, Volkmar FR (2008). Production of syllable stress in speakers with autism spectrum disorders. Res. Autism Spectr. Disord..

[13] Patel SP (2020). An acoustic characterization of prosodic differences in autism spectrum disorder and first-degree relatives. J. Autism Dev. Disord..

[14] Gregory S (1990). Analysis of fundamental frequency reveals covariation in interview partners’ speech. J. Nonverbal Behav..

[15] Levitan, R. *et al.* Acoustic-prosodic entrainment and social behavior. in *Proceedings of the 2012 Conference of the North American Chapter of the Association for Computational Linguistics: Human language technologies* 11–19 (2012).

[16] Brennan SE, Clark HH (1996). Conceptual pacts and lexical choice in conversation. J. Exp. Psychol. Learn. Mem. Cogn..

[17] Allen ML, Haywood S, Rajendran G, Branigan H (2011). Evidence for syntactic alignment in children with autism. Dev. Sci..

[18] Branigan HP, Pickering MJ, Pearson J, McLean JF, Brown A (2011). The role of beliefs in lexical alignment: Evidence from dialogs with humans and computers. Cognition.

[19] Garrod S, Anderson A (1987). Saying what you mean in dialogue: A study in conceptual and semantic co-ordination. Cognition.

[20] Kruyt J, Beňuš Š (2021). Prosodic entrainment in individuals with autism spectrum disorder. Top. Linguist..

[21] Landa R (1992). Social language use in parents of autistic individuals. Psychol. Med..

[22] Losh M (2012). Defining genetically meaningful language and personality traits in relatives of individuals with fragile X syndrome and relatives of individuals with autism. Am. J. Med. Genet. B. Neuropsychiatr. Genet..

[23] Losh M, Childress D, Lam K, Piven J (2008). Defining key features of the broad autism phenotype: A comparison across parents of multiple- and single-incidence autism families. Am. J. Med. Genet. Part B Neuropsychiatr. Genet. Off. Publ. Int. Soc. Psychiatr. Genet..

[24] Braff DL (2015). The importance of endophenotypes in schizophrenia research. Schizophr. Res..

[25] Kendler KS, Neale MC (2010). Endophenotype: A conceptual analysis. Mol. Psychiatry.

[26] Walters JTR, Owen MJ (2007). Endophenotypes in psychiatric genetics. Mol. Psychiatry.

[27] Almasy L (1999). Human pedigree-based quantitative-trait-locus mapping: Localization of two genes influencing HDL-cholesterol metabolism. Am. J. Hum. Genet..

[28] Williams RB (2010). Central nervous system serotonin and clustering of hostility, psychosocial, metabolic, and cardiovascular endophenotypes in men. Psychosom. Med..

[29] Mitchell BD (1999). Diabetes and hypertension in Mexican American families: Relation to cardiovascular risk. Am. J. Epidemiol..

[30] Gur RE (2006). The consortium on the genetics of schizophrenia: Neurocognitive endophenotypes. Schizophr. Bull..

[31] Gottesman II, Shields J (1972). Schizophrenia and Genetics.

[32] Bolton P (1994). A case–control family history study of autism. J. Child Psychol. Psychiatry..

[33] Piven J (1997). Personality and language characteristics in parents from multiple-incidence autism families. Am. J. Med. Genet..

[34] Frazier TW (2015). Quantitative autism symptom patterns recapitulate differential mechanisms of genetic transmission in single and multiple incidence families. Mol. Autism.

[35] Whitehouse A, Coon H, Miller J, Salisbury B, Bishop DVM (2010). Narrowing the broader autism phenotype: A study using the Communication Checklist-Adult Version (CC-A). Autism.

[36] Lindgren KA, Folstein SE, Tomblin JB, Tager-Flusberg H (2009). Language and reading abilities of children with autism spectrum disorders and specific language impairment and their first-degree relatives. Autism Res..

[37] Patel SP, Kim JH, Larson CR, Losh M (2019). Mechanisms of voice control related to prosody in autism spectrum disorder and first-degree relatives. Autism Res..

[38] Russo N, Larson CR, Kraus N (2008). Audio-vocal system regulation in children with autism spectrum disorders. Exp. Brain Res..

[39] Gambi C, Pickering MJ (2013). Prediction and imitation in speech. Front. Psychol..

[40] Wynn CJ, Borrie SA, Sellers TP (2018). Speech rate entrainment in children and adults with and without autism spectrum disorder. Am. J. Speech Lang. Pathol..

[41] Bone D (2014). The psychologist as an interlocutor in autism spectrum disorder assessment: Insights from a study of spontaneous prosody. J. Speech Lang. Hear. Res..

[42] Neely JH (1977). Semantic priming and retrieval from lexical memory: Roles of inhibitionless spreading activation and limited-capacity attention. J. Exp. Psychol. Gen..

[43] Branigan HP, Tosi A, Gillespie-Smith K (2016). Spontaneous lexical alignment in children with an autistic spectrum disorder and their typically developing peers. J. Exp. Psychol. Learn. Mem. Cogn..

[44] Hopkins Z, Yuill N, Branigan HP (2017). Inhibitory control and lexical alignment in children with an autism spectrum disorder. J. Child Psychol. Psychiatry.

[45] Toichi M, Kamio Y (2001). Verbal association for simple common words in high-functioning autism. J. Autism Dev. Disord..

[46] Harper-Hill K, Copland D, Arnott W (2014). Efficiency of lexical access in children with autism spectrum disorders: Does modality matter?. J. Autism Dev. Disord..

[47] Henderson LM, Clarke PJ, Snowling MJ (2011). Accessing and selecting word meaning in autism spectrum disorder. J. Child Psychol. Psychiatry.

[48] Kamio Y, Toichi M (2000). Dual access to semantics in autism: Is pictorial access superior to verbal access?. J. Child Psychol. Psychiatry.

[49] Kamio Y, Robins D, Kelley E, Swainson B, Fein D (2007). Atypical lexical/semantic processing in high-functioning autism spectrum disorders without early language delay. J. Autism Dev. Disord..

[50] Slocombe KE (2013). Linguistic alignment in adults with and without Asperger’s syndrome. J. Autism Dev. Disord..

[51] Losh M, Capps L (2003). Narrative ability in high-functioning children with autism or Asperger’s syndrome. J. Autism Dev. Disord..

[52] Lord C (2012). Autism Diagnostic Observation Schedule.

[53] Wechsler, D. *Wechsler Abbreviated Scale of Intelligence *(*WASI*) (1999).

[54] Wechsler D (2003). Wechsler Intelligence Scale for Children.

[55] Reichel, U. & Cole, J. Entrainment analysis of categorical intonation representations. in *Proceedings of Phonetics & Phonology* 165–168 (2016).

[56] ELAN. (2017).

[57] Carletta J (1997). The reliability of a dialogue structure coding scheme. Comput. Linguist..

[58] Reichel, U. CoPaSul Manual—Contour-based parametric and superpositional intonation stylization (2016).

[59] Heinrich C, Schiel F (2014). The influence of alcoholic intoxication on the short-time energy function of speech. J. Acoust. Soc. Am..

[60] Cole, J., Roettger, T., Reichel, U. & Mády, K. Prosodic entrainment in dialog acts (2018).

[61] Kassambara, A. & Mundt, F. Package ‘factoextra.’ extract and visualize the results of multivariate data analyses (2016).

[62] Raiche, G. nFactors: An R package for parallel analysis and non-graphical solutions to the Cattell scree tests (2010).

[63] Duran ND, Paxton A, Fusaroli R (2019). ALIGN: Analyzing linguistic interactions with generalizable techNiques—A Python library. Psychol. Methods.

[64] Mikolov, T., Chen, K., Corrado, G. & Dean, J. Efficient estimation of word representations in vector space. arXiv:1301.3781 (2013).

[65] Bates, D., Mächler, M., Bolker, B. & Walker, S. Fitting linear mixed-effects models using lme4. arXiv:1406.58231 (2014).

[66] Healey PGT, Purver M, Howes C (2014). Divergence in dialogue. PLoS One.

[67] Levitan, R. *et al.* Implementing acoustic-prosodic entrainment in a conversational avatar. in *Proceedings of Interspeech* 1166–1170 (2016).

[68] Schweitzer, A. & Walsh, M. Exemplar dynamics in phonetic convergence of speech rate. in *Proceedings of Interspeech* (2016).

[69] Beňuš Š (2014). Social aspects of entrainment in spoken interaction. Cognit. Comput..

[70] Michalsky, J., Schoormann, H. & Schultze, T. Towards the prosody of persuasion in competitive negotiation. The relationship between f0 and negotiation success in same sex sales tasks. in *Proceedings of Interspeech* 311–315 (2019).

[71] Filipe MG, Frota S, Castro SL, Vicente SG (2014). Atypical prosody in Asperger syndrome: Perceptual and acoustic measurements. J. Autism Dev. Disord..

[72] Tsuang MT, Bar JL, Stone WS, Faraone SV (2004). Gene–environment interactions in mental disorders. World Psychiatry.

[73] Lehnert-LeHouillier H, Terrazas S, Sandoval S (2020). Prosodic entrainment in conversations of verbal children and teens on the autism spectrum. Front. Psychol..

[74] DePape A-MR, Chen A, Hall GBC, Trainor LJ (2012). Use of prosody and information structure in high functioning adults with autism in relation to language ability. Front. Psychol..

[75] Menshikova, A., Kocharov, D. & Kachkovskaia, T. Lexical Entrainment and Intra-Speaker Variability in Cooperative Dialogues. in *Interspeech 2021* 1957–1961 (ISCA, 2021). 10.21437/Interspeech.2021-1441

[76] Sedgewick F, Hill V, Yates R, Pickering L, Pellicano E (2016). Gender differences in the social motivation and friendship experiences of autistic and non-autistic adolescents. J. Autism Dev. Disord..

[77] Van Wijngaarden-Cremers PJM (2014). Gender and age differences in the core triad of impairments in autism spectrum disorders: A systematic review and meta-analysis. J. Autism Dev. Disord..

[78] Parish-Morris J (2017). Linguistic camouflage in girls with autism spectrum disorder. Mol. Autism.

[79] Crompton CJ (2020). Neurotype-matching, but not being autistic, influences self and observer ratings of interpersonal rapport. Front. Psychol..

[80] Morrison KE (2020). Outcomes of real-world social interaction for autistic adults paired with autistic compared to typically developing partners. Autism.

[81] Rifai OM, Fletcher-Watson S, Jiménez-Sánchez L, Crompton CJ (2021). Investigating markers of rapport in autistic and nonautistic interactions. Autism Adulthood.

