# Supplementary material for: Verbal entrainment in autism spectrum disorder and first-degree relatives
Source: Sci Rep. 2022 Jul 7;12:11496. doi: 10.1038/s41598-022-12945-4 (PMC9262979; doi:10.1038/s41598-022-12945-4)
Supplement: Supplementary file 1 — Supplementary Information. [file 41598_2022_12945_MOESM1_ESM.docx]

Supplementary Methods

**Social Communication and Within-Family Associations**

Social communication skills were assessed to investigate how disrupted entrainment might relate to the ASD language phenotype and subclinical language-related variation in biological relatives. Social communication skills in the ASD and ASD Control groups were assessed using the Pragmatic Rating Scale-School Age (PRS-SA)^97^. The PRS-SA was rated using video recordings of the participant’s conversation and interaction with the examiner during the ADOS-2^66^. Each interaction was independently rated by two coders blind to group using a three-point scale (0: absent; 1: mildly present; 2: present). Discrepancies between coders were resolved via discussion, and the consensus scores were used in analyses. In addition to an overall total score of pragmatic language ability, the PRS-SA provides six subscales based on individual items. The subscales include: presupposition (e.g., redundant conversation, inadequate clarification); discourse management (e.g., acknowledgement; reciprocal conversation; response elaboration); speech/language behaviors that may impact pragmatic language (e.g., overly formal language; scripted speech); suprasegmentals (e.g., intonation modulation; rate of speech; volume modulation); and nonverbal communication (e.g., gestures; eye-contact; facial expressions).

Social communication skills in the ASD Parent and Parent Control groups were assessed using the Pragmatic Rating Scale (PRS)^21^. The PRS was rated based on video recordings of a semi-structured interview between each parent and the examiner. The interview includes questions about the parent’s early childhood, schooling, friendships/relationships, and occupations. The PRS was scored using the same three-point scale and consensus procedure described above for the PRS-SA. Similar to the PRS-SA, the PRS provides three subscales in addition to a total score. The PRS subscales include: dominant conversation style (e.g., too detailed; tangential); listener expectation (e.g., failure to reciprocate); and suprasegmentals (intonation modulation; rate of speech; volume modulation).

Statistical Analysis

Pearson correlations were conducted to assess associations between measures of verbal entrainment and measures of social communication in the ASD and ASD Control groups combined and the parent groups combined. Additionally, Pearson correlations were conducted to assess familiality of verbal entrainment across mother-child dyads in the ASD (n = 17) and control (n = 19) groups. Father-child correlations were not examined due to limited sample sizes. Correlations were not corrected for multiple comparisons to transparently present patterns that may be useful in informing future work. Relationships should therefore be interpreted with caution and in consideration of the strength of associations.

Supplementary Results

**Relationships Between Verbal Entrainment and Social Communication Skills**

In the ASD and ASD Control groups combined, increased pragmatic (i.e., social) language violations were associated with poorer *prosodic* (F0 at the dialog act unit factor 1: r = .32, p = .04), *semantic* (r = -.31, p = .04; Figure 4), and *syntactic* (r = -.59, p < .001) entrainment. No such associations were evident in the parent groups combined (rs < .06, ps > 0.09).

**Familiality of Verbal Entrainment**

Pearson correlations between ASD mother-child dyads revealed positive associations between *prosodic* entrainment of pitch/F0 at the salient syllable level (factor 2: r = .50, p = .04) and *lexical* entrainment (r = .51, p = .04) in ASD families. Associations between mother-child dyads in control families revealed positive associations in *prosodic* entrainment, specifically F0 at the salient syllable (factor 1: r = .52, p = .02) and rhythmic entrainment (factor 2: r = .58, p = .01.

**Supplementary Discussion**

Associations between entrainment and a comprehensive measure of pragmatic skill in conversation emerged across several domains of verbal entrainment in the ASD group, likely reflecting a complex interrelationship, where core pragmatic skills impacted in ASD can both contribute to and be reciprocally impacted by differences in entrainment. Interestingly, the strongest relationships between verbal entrainment and pragmatic language skills emerged in the domain of syntax, where entrainment was not evident. Given the lack of syntactic entrainment across the ASD and ASD Control groups, this finding may reflect a general association between reduced language complexity and greater difficulty in pragmatic language skills. Alternatively, associations may reflect the impact a lack of syntactic entrainment or disentrainment has on broader pragmatic language abilities. Considering evidence of both syntactic entrainment and disentrainment as supporting communicative effectiveness, a lack of either pattern may potentially hinder overall pragmatic language abilities. Relationships with prosodic and semantic entrainment, however, suggest that the inability to entrain to one’s partner at lower levels of the linguistic hierarchy impedes pragmatic language, or higher-level, success. For instance, reduced semantic entrainment may manifest as increased topic perseveration or difficulty maintaining a topic, both of which are pragmatic difficulties that are prominent in ASD. Furthermore, in addition to overlapping differences in prosodic entrainment among the ASD and ASD Parent groups, mother-child associations revealed familiality of prosodic entrainment, suggest that prosody may be an important marker of biological contributions to social communication differences across developmental disorders, like ASD.

Supplementary Table 1. Variables included in exploratory factor analyses (EFAs) examining prosodic entrainment. Variables at the Dialog Act Unit level correspond to ‘global’ measures in the CoPaSul feature set, while variables at the Salient Syllable level correspond to CoPaSul ‘local’ measures. The Salient Syllable unit is automatically detected based on energy and duration thresholds tuned to distinguish syllables with phrasal stress prominence^98^. F0 variables of baseline, midline and topline variables are calculated in each window of 200 ms, positioned every 10 ms across the dialog act or salient syllable unit. The baseline is the median of the lower 10^th^ percentile of F0 values within the analysis window; the topline is the upper 10^th^ percentile, and the mean is the mean of all F0 values in the window. F0 RMSD variables are calculated in each window of 300 ms as the root-mean-square deviation from the mean over all F0 values, or over the baseline, topline, midline and range values in the analysis window. Rhythm measures are calculated in CoPaSul following Heinrich & Schiel^73^, using a discrete cosine transform on the energy contour and calculating the influence of the stressed syllable on this contour as the relative weight of the coefficients below a rate of approximately 10Hz.

|  | **Variable** | **Description** |
| --- | --- | --- |
| **F0 at the Dialog Act Unit Level** | F0 mean | mean F0 in dialog act |
|  | F0 max | max F0 in dialog act |
|  | F0 standard deviation | standard deviation of F0 in dialog act |
|  | baseline mean | Mean bottom of the F0 range across dialog act. |
|  | baseline slope | Slope of F0 baseline across dialog act |
|  | midline mean | Mean middle of the F0 range across dialog act |
|  | midline slope | Slope of F0 midline across dialog act |
|  | topline mean | Mean top of the F0 range across dialog act |
|  | topline slope | Slope of F0 topline across DA unit |
|  | range mean | Mean of F0 range (topline-baseline) across dialog act |
|  | range rate | Rate of declination of F0 range (Hz or semitone per second) across dialog act |
| **F0 at the Salient Syllable Level** | F0 mean | mean F0 in salient syllable |
|  | F0 max | max F0 in salient syllable |
|  | F0 standard deviation | standard deviation of F0 in dialog act |
|  | pooled RMSD | Mean deviation in F0 in salient syllable |
|  | baseline RMSD | Mean deviation in F0 baseline in salient syllable |
|  | midline RMSD | Mean deviation in F0 midline in salient syllable |
|  | topline RMSD | Mean deviation in F0 topline in salient syllable |
|  | range RMSD | Mean deviation in F0 range in salient syllable |
| **Rhythm at the Dialog Act Unit Level** | salient syllable influence on energy | Influence of prominent (stressed) syllables on the energy contour of the dialog act |
|  | salient syllable rate | Prominent (stressed) syllable rate in dialog act |
|  | syllable influence on energy | Influence of (stressed or unstressed) syllables on energy contour of the dialog act |
|  | syllable rate | (All) Syllable rate in dialog act |

Supplementary Table 2. Factor loadings for confirmatory factor analyses (CFAs) examining prosodic entrainment.

**Supplementary Table 3.** Statistical findings for prosodic entrainment.

**Supplementary Table 4.** Statistical findings for lexical, syntactic, and semantic entrainment.
